# Remote Manipulation of Droplets on a Flexible Magnetically Responsive Film

**DOI:** 10.1038/srep17843

**Published:** 2015-12-09

**Authors:** Jeong Hun Kim, Seong Min Kang, Byung Jun Lee, Hangil Ko, Won-Gyu Bae, Kahp Yang Suh, Moon Kyu Kwak, Hoon Eui Jeong

**Affiliations:** 1 School of Mechanical and Aerospace Engineering, Seoul National University, Seoul 151-742, Republic of Korea; 2 Department of Mechanical Engineering, Ulsan National Institute of Science and Technology (UNIST), Ulsan 689-798, Republic of Korea; 3 Department of Mechanical Engineering, Kyungpook National University, Daegu 702-701, Republic of Korea

## Abstract

The manipulation of droplets is used in a wide range of applications, from lab-on-a-chip devices to bioinspired functional surfaces. Although a variety of droplet manipulation techniques have been proposed, active, fast and reversible manipulation of pure discrete droplets remains elusive due to the technical limitations of previous techniques. Here, we describe a novel technique that enables active, fast, precise and reversible control over the position and motion of a pure discrete droplet with only a permanent magnet by utilizing a magnetically responsive flexible film possessing actuating hierarchical pillars on the surface. This magnetically responsive surface shows reliable actuating capabilities with immediate field responses and maximum tilting angles of ~90°. Furthermore, the magnetic responsive film exhibits superhydrophobicity regardless of tilting angles of the actuating pillars. Using this magnetically responsive film, we demonstrate active and reversible manipulation of droplets with a remote magnetic force.

The manipulation of droplets is of significant interest for a broad range of applications from lab-on-a-chip devices for biological and chemical analyses to bioinspired functional surfaces. To manipulate a droplet, micro- or nanopatterned surfaces have been suggested as a passive approach, in which unique structural features (e.g., slanted structure) or chemical gradients enable liquid wetting and spreading in a specific direction^1^,^2^,^3^,^4^,^5^. Although these approaches enable control over liquid wetting and spreading without external forces, they are typically slow and not reversible, presenting inherent limitations to the passive mechanisms. A set of active droplet manipulation techniques have also been proposed, including electrowetting^6^,^7^, dielectrophoresis^8^,^9^, surface acoustic waves and thermocapillary force^10^,^11^. Compared with the passive approaches, these active techniques provide enhanced control over droplet position and motion in a fast and reliable fashion. In particular, the electrowetting technique has demonstrated its versatility in microfluidic systems, as it enables precise manipulation of discrete droplets through a programmed path. Nonetheless, the electrowetting platform requires electrodes formed on a substrate, as well as an external power source, for the manipulation of liquid droplets, which limits the scalability and applicability of the technique.

To this end, magnetically actuating surfaces with micro- or nanoscale structures have considerable potential to be used for the active and dynamic manipulation of droplets because of their reversible and instantaneous structural tunability in response to a remote and non-destructive magnetic field. The structural changes of micro- or nanostructures upon the application of a magnetic force can be utilized for the direct control of liquid droplets. However, previous droplet manipulation techniques based on magnetic force are mostly limited to irreversible transitions of wetting states without the ability to control the position and motion of discrete droplets^12^,^13^,^14^. Although some studies reported positional control of droplets based on using a magnetic field, they utilized a droplet mixed with magnetic nanoparticles^15^,^16^,^17^, which significantly limits broad application of the techniques. Here, we report a novel technique that enables active and dynamic control over the position and motion of a pure discrete droplet by utilizing a magnetically responsive flexible film comprising reversibly actuating hierarchical pillars on the surface. In this approach, a discrete droplet of water can be rapidly manipulated to arbitrary target locations on the flexible film with only the use of a permanent magnet without the need for predefined electrodes or mixing droplets with magnetic particles. The flexible film with actuating hierarchical pillars is simply fabricated by a mouldless self-assembly of a solution comprising precured polymers and magnetic particles under a magnetic field. This magnetically responsive surface exhibits reliable actuating capabilities with immediate field responses and maximum tilting angles of ~90°. Furthermore, the magnetic responsive film exhibits superhydrophobicity regardless of the tilting angles of the actuating pillars. Using this novel magnetically responsive film with a superhydrophobic nature, we demonstrate highly precise and dynamic manipulation of a discrete droplet in an active and instant manner using only a permanent magnet.

## Results

### Preparation of magnetically responsive film

**Figure 1:**
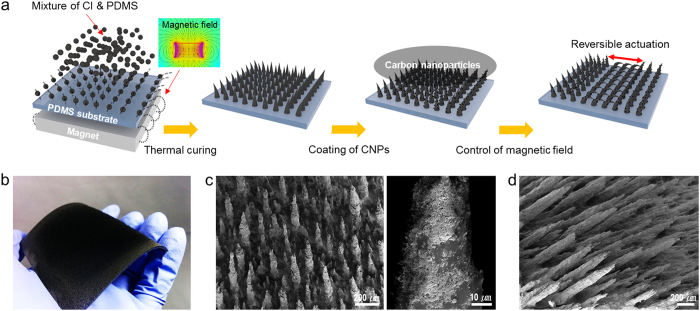
Magnetically responsive film with dynamically actuating hierarchical pillar arrays. (**a**) A schematic illustration of the fabrication procedure of the magnetically responsive film. A solution of magnetic particles and PDMS sprayed on a cured PDMS substrate spontaneously arranges along the direction of the magnetic field and forms pillar-like structures. Subsequent thermal curing of the sprayed samples fixes the field-aligned pillar shapes. Coating of carbon nanoparticles (CNPs) over the arrays results in magnetically actuating hierarchical pillars with superhydrophobicity. (**b**) A photograph of the fabricated large area magnetically responsive flexible film. (**c**) Scanning electron microscopy (SEM) images of the magnetically responsive hierarchical pillar array. (**d**) SEM image of the hierarchical pillar array bent in response to a remote magnetic force.

Among many possible approaches to generate dynamically tuneable structures, magnetically actuated surfaces are particularly attractive due to their instantaneous response and remote controllability and the nondestructive nature of magnetic fields^18^. Figure 1a depicts in detail the procedure for the preparation of a magnetically responsive film with self-assembled hierarchical pillar arrays on the surface. First, a solution of magnetic particles and polydimethylsiloxane (PDMS) was prepared by adding carbonyl iron (CI) particles into a mixture of uncured PDMS and hexane. Then, 1.5 ml of the solution was sprayed using a spray gun onto a cured PDMS substrate that was placed on a neodymium magnet. Then, the mixture of ferromagnetic CI particles and uncured PDMS spontaneously arranged along the direction of the magnetic field and formed pillar-like structures. Subsequent thermal curing of the sprayed samples fixed the field-aligned pillar shapes, resulting in composite pillar arrays made of PDMS and magnetic particles over a large area; the magnetic particles in the pillars enabled dynamic tuning of structural motions, whereas the polymeric matrix defined the structural geometry (Fig. 1b,d). The coating of carbon nanoparticles (CNPs) over the arrays resulted in magnetically actuating hierarchical pillar arrays with superhydrophobicity. Previous studies have shown that well-defined magnetically responsive structures can be generated via photolithographic or soft lithographic techniques, in which pre-defined masks or moulds were applied to precured polymers mixed with magnetic particles^14^,^19^,^20^. Although these methods are useful to fabricate magnetically responsive surfaces with ordered micro- or nanoscale structures, additional lithographic processes to prepare the mask or mould are required. Additionally, instantaneous modulation of geometries of magnetically responsive structures is not possible with these mould-based approaches, because the geometries of the resulting samples are usually limited by the patterns of the masks or moulds.

**Figure 2:**
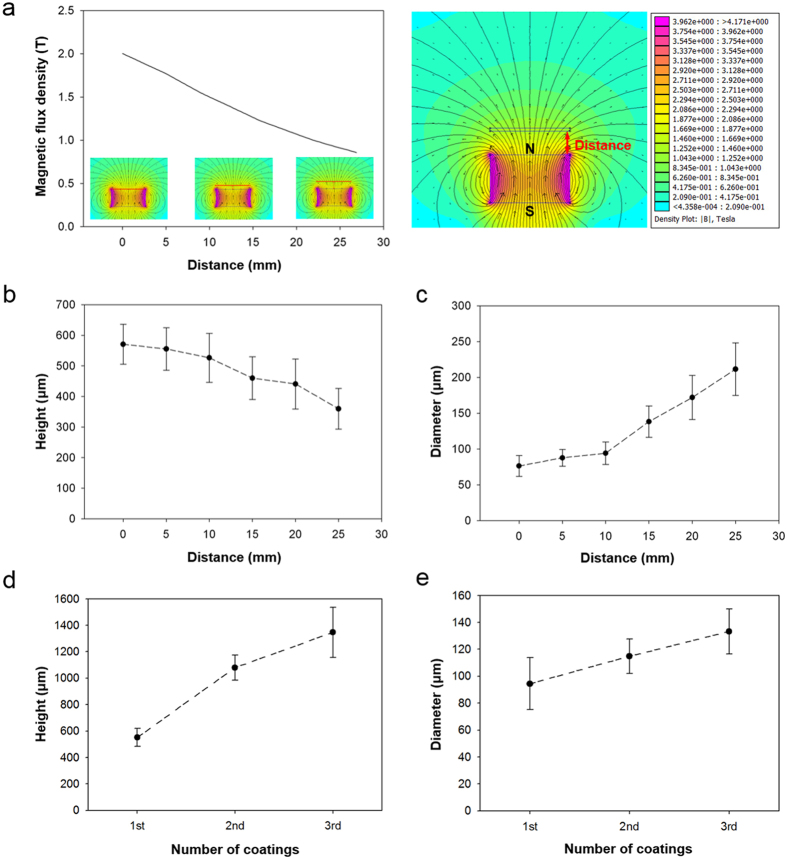
Modulation of pillar geometries by controlling the magnetic field and number of spray coating-curing processes. (**a**) Magnetic flux density as a function of distance between the magnet and the sample. (**b**) Averaged height and (**c**) diameter of the fabricated magnetically responsive pillars as a function of distance between the magnet and the sample. (**d**) Averaged height and (**e**) diameter of the fabricated magnetically responsive pillars as a function of the number of coating-curing processes.

By contrast, pillar geometries can be simply tuned in our approach by the modulation of a magnetic field. As shown in Fig. 2a (and Fig. S1), the magnetic flux density varies with the distance between the substrate and the magnet. Therefore, pillar arrays with different diameters and heights can be generated by controlling the distance between the substrate and the magnet during the fabrication procedure (Fig. S2). For example, when the substrate is placed on the magnet without a gap between them, the average height of the micropillars was maximized to ~580 μm. By increasing the distance between the substrate and magnet, the height was decreased and reached ~380 μm at a separation of 25 mm (Fig. 2b). By contrast, the diameter of the pillar arrays was reduced to ~70 μm when the separation was zero and monotonically increased with the distance, reaching ~210 μm at a distance of ~25 mm (Fig. 2c). This is because the solution containing magnetic particles experiences higher magnetic field density and strength when the separation is minimized. The height and diameter of the pillar arrays can also be modulated by repeating the process of spray coating-curing (Fig. S3). When the coating-curing process was performed only once, the height and the diameter of the pillars were ~500 μm and ~90 μm, respectively. During this process, the distance between the sample and the magnet was maintained at zero, and the amount of mixture sprayed in a single coating was ~1.5 ml. By repeating this process, the height and diameter were increased, as shown in Fig. 2d,e. After the third coating and curing process, the height reached ~1300 μm and the diameter was increased to ~130 μm, generating pillar structures with a very high aspect ratio (>10). The pillar geometry can also be adjusted by controlling the amount of sprayed mixture on the PDMS substrate (Fig. S4). When we sprayed 0.5 ml of the mixture, a pillar array with relatively low density and height was generated. When we increased the amount during spraying, micropillar arrays with higher density and height could be generated. The generated micropillars with controllable geometries can be further modified into micro- and nanoscale combined hierarchical pillar arrays by coating the micropillars with CNPs, which provide the array with thermodynamically stable superhydrophobicity. With simple tunability of the structural geometries, including diameter, height, and hierarchy, of the pillar arrays, our approach provides a novel strategy for generating magnetically responsive hierarchical pillar arrays with robust superhydrophobicity over a large area by simple self-assembly without the need for complex fabrication processes or predefined moulds. Furthermore, under same fabrication conditions, our approach showed a robust reproducibility as demonstrated in Fig. 2b–d.

### Active control of the dynamic structural change of the magnetic pillar arrays by an external magnetic field

**Figure 3:**
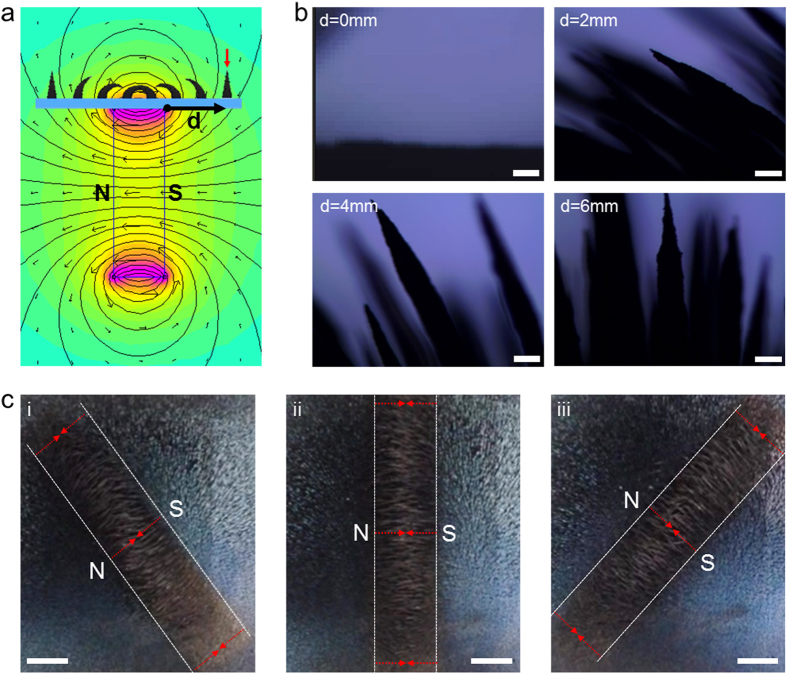
Dynamic structural control of the magnetically responsive pillar array with a permanent magnet. (**a**) Simulation of magnetic flux density around the pillar array when a magnet is placed under the sample. (**b**) Side views of the dynamic response of the pillar array when the magnet approaches the targeted pillars (red arrows in Fig. 3a) at a horizontal distance “d” from the edge of the magnet. Scale bars are 200 μm. (**c**) Top-views of the magnetically responsive pillar array placed on a rectangular magnet. The pillars located directly above the magnet are significantly bent. The region of bent pillars rotates along with the magnet. Scale bars are 1 cm.

To investigate the dynamic response of the pillar arrays under a magnetic field, structural changes of the pillar were observed with upright optical microscopy by changing the horizontal position of a rectangular neodymium magnet under the films. When the rectangular magnet is placed under the film, the magnetic flux density is maximized at the centre and rapidly decreased with the distance from the centre (Fig. 3a). Consequently, pillars located directly above the magnet undergo the strongest magnetic field, whereas pillars located away from the boundaries of the magnet are under little influence of the field. This indicates that the bending and actuating behaviours of the array can be simply and precisely controlled by changing the location of the magnet under the sample. For example, when we placed a rectangular magnet (2 cm (W) × 2 cm (L) × 1 cm (T), and the maximum flux density: ~3 T) 6 mm away from the south or north pole face of the magnet (defined as “d” in Fig. 3a), the pillars maintained their original vertical position without any bending (Fig. 3b). However, as the magnet approached the sample, the pillar arrays started to respond strongly to the magnet because of the highly ferromagnetic carbonyl iron. When the distance was ~4 mm, the array bent to a tilting angle of ~60°. As the distance decreased, the magnetic flux density influence on the pillar array increased, resulting in more bending. Furthermore, the pillar array was nearly flattened to the surface with tilting angles of ~90° when the horizontal distance between the side of the magnet and the sample was zero. When the magnet was removed, the arrays immediately returned to their original vertical position by the elastic restoring force of the PDMS matrix (see movie S1 and Fig. S5), demonstrating fast and reliable actuating, as well as large bending capability, in response to a remote magnetic force. Figure 3c shows top views of the magnetically responsive pillar array placed over a rectangular magnet. As shown, only the pillars located directly above the magnet were significantly bent, whereas pillars beyond the magnet’s face showed little or no bending. The pillars located at the edges of the magnet were bent towards the centre of the side face of the magnet along the magnetic field formed between the north (N) and the south (S) pole faces of the magnet. When we rotate the magnet under the film, the direction of the bent pillars also changed along with the magnet (Fig. 3c).

### Wetting properties of the magnetically responsive film

**Figure 4:**
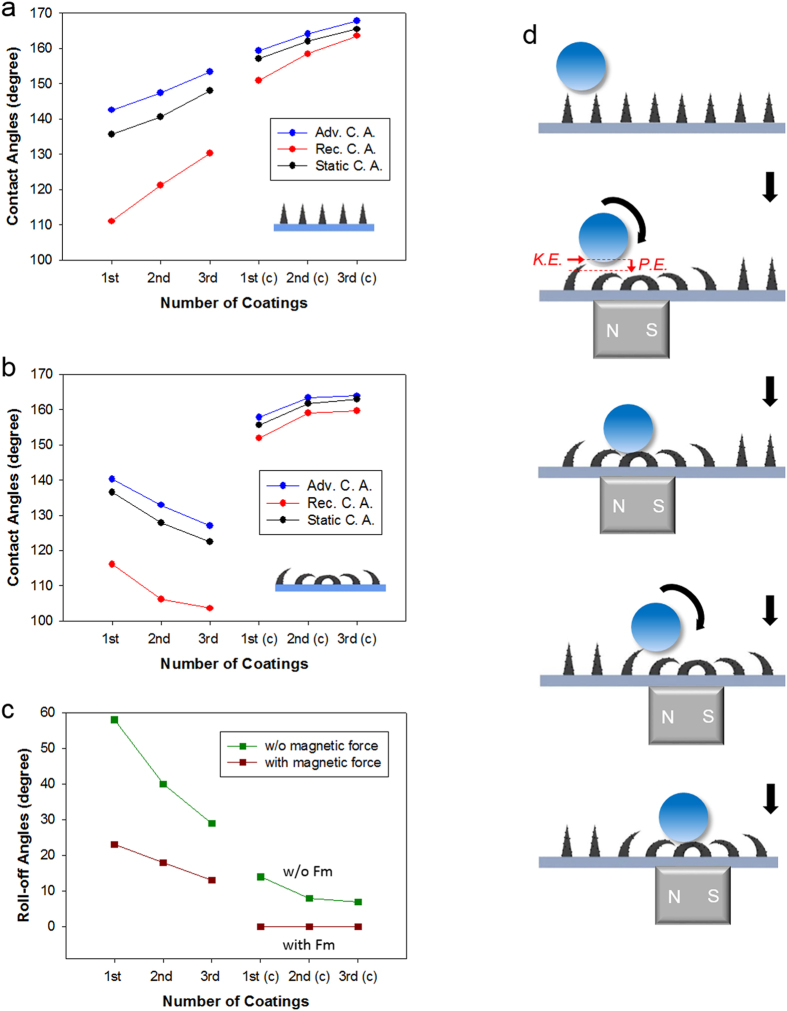
Analysis of the wetting properties of the magnetically responsive pillar arrays. (**a**) Contact angles of DI water on various magnetically responsive pillar arrays with different numbers of coating-curing processes when no magnetic field is applied to the sample. (**b**) Contact angles of DI water on various magnetically responsive pillar arrays with different numbers of coating-curing processes when the pillar arrays are fully flattened on the substrate by an applied magnetic field. (**c**) Roll-off angles of DI water on various magnetically responsive pillar arrays for different numbers of coating-curing processes and magnetic field application. The symbol “(**c**)” on the x-axis of the graphs in (**a–c**) represents CNP coated samples. (**d**) Schematic illustration of active droplet manipulation on the magnetically actuating hierarchical pillar array. When the pillars are bent by a magnetic force, a local difference in the potential energy (“PE”) is generated in the array. Furthermore, actuation of the pillars provides a driving force (kinetic energy, “KE”) for the droplet to move along the direction of the pillars. As a result, the droplet moves towards the location of the bent pillars.

The surface wetting properties of the arrays related to the CNP coating and the number of coating-curing processes were investigated. The CNP coating adds structural hierarchy to the pillar arrays, and the repetition of the spray coating-curing process increases the diameter and the height of the pillar arrays, as described above. For pillar arrays in their original unbent configuration, arrays with more coating-curing processes exhibited higher contact angles (CA) and reduced contact angle hysteresis (CAH) (Fig. 4a). This is because the air fraction under the droplet is increased for the samples with more spray coating-curing processes (see Fig. S6). Furthermore, coating of the array with CNPs significantly enhanced CA > 150° and reduced CAH < 10°, enabling superhydrophobic wetting by introducing dual roughness to the array^21^,^22^,^23^.

When the pillar arrays were bent under a magnetic field, different wetting behaviours were observed depending on the presence of CNPs on the arrays. For example, arrays without CNPs showed decreased CAs with increasing spray coating-curing processes (Fig. 4b). CAs on the arrays prepared with a third coating process reduced to ~123°. Additionally, the bent pillar arrays without CNPs showed a relatively high CAH of over 23°, which could hinder the manipulation of droplets on the arrays. This is due to the increase in solid fraction under the droplet for samples with more coating-curing processes when the arrays without CNPs are bent under a magnetic field (see Fig. S7). By contrast, pillar arrays with CNP layers showed enhanced CAs with increasing coating-curing processes (Fig. 4b). Interestingly, these arrays maintained their superhydrophobicity (CAs >150 ° and CAH<10 °) even when the arrays were bent by a magnetic force (Fig. 4b). This is because the CNP coating creates nanoscale roughness over the surface of the micropillars, resulting in micro- and nanoscale combined hierarchical structures. These hierarchical architectures enable the array to maintain reduced contact with the droplet and thus a superhydrophobic wetting state regardless of the bending angle of the pillar arrays. These results indicate that a discrete droplet would not wet these self-assembled hierarchical pillar arrays even when the arrays are actuating under a magnetic force, which may enable active and reversible manipulation of a droplet on the pillar arrays. Although previous studies have reported droplet manipulation based on magnetic force, they are mostly limited to demonstrating the transition of the wetting state from the Cassie-Baxter state to the Wenzel state upon actuation or bending of micro- or nanoscale structures. In this case, not only is the wetting transition irreversible, but precise and fast control of the position and motion of discrete droplets is also not possible^12^,^13^,^14^. By contrast, our results demonstrate a strong possibility of rapid and reversible control over the position and motion of droplets using magnetically actuated surfaces.

To further characterize the wetting properties, we also measured the roll-off angles (ROAs) of a droplet on the pillar array, which were determined as the tilting angles of the substrate at which the droplet starts to roll off^24^. The ROA of a vertical pillar array without CNPs fabricated with a single coating-curing process was ~59°. The ROA decreased to ~30° for triple coated samples. By coating CNPs on the pillars, the ROA dramatically decreased to ~17° for single coated samples and further decreased to <10° for triple coated samples (Fig. 4c). Interestingly, when we applied a magnetic field to the samples, the ROAs of the pillar arrays were greatly reduced. The ROAs of the pillars without CNPs were 22°, 18° and 12°, respectively, with increasing spray coating-curing processes. Strikingly, pillar arrays coated with CNPs shows ROAs of ~0° when bending under a magnetic force. This means that these arrays have a strong potential to transport and manipulate a liquid droplet on the surface with only a remote magnetic field and without the need for inclining the substrate. It is noted that the ROAs of DI water on the CNP-coated pillars were ~0° in magnetic field with any number of CNP coatings. Theoretically, the ROAs should be affected by the CAH on the same surfaces. However, in this work, the ROAs showed ~0° regardless of existence of CAH due to the potential energy caused by bending of magnetically responsive pillar arrays. Figure 4d shows an illustration of droplet manipulation on our flexible magnetically responsive film. When pillars above the magnet are bent, a local difference in the potential energy is generated in the array. Furthermore, actuation of the pillars provides a driving force for the droplet to move along the tilting direction of the pillars. As a result, the droplet moves towards the location of the bent pillars. The pillars in the places where the magnet passed recovered their original vertical shape, whereas the magnet bent pillars further down the path. This series of subsequent local actuating of pillar arrays by controlling a magnetic field enabled precise and active manipulation of the droplet (See Figure S8 and Movie S2).

### Remote manipulation of droplets on the magnetically responsive film

**Figure 5:**
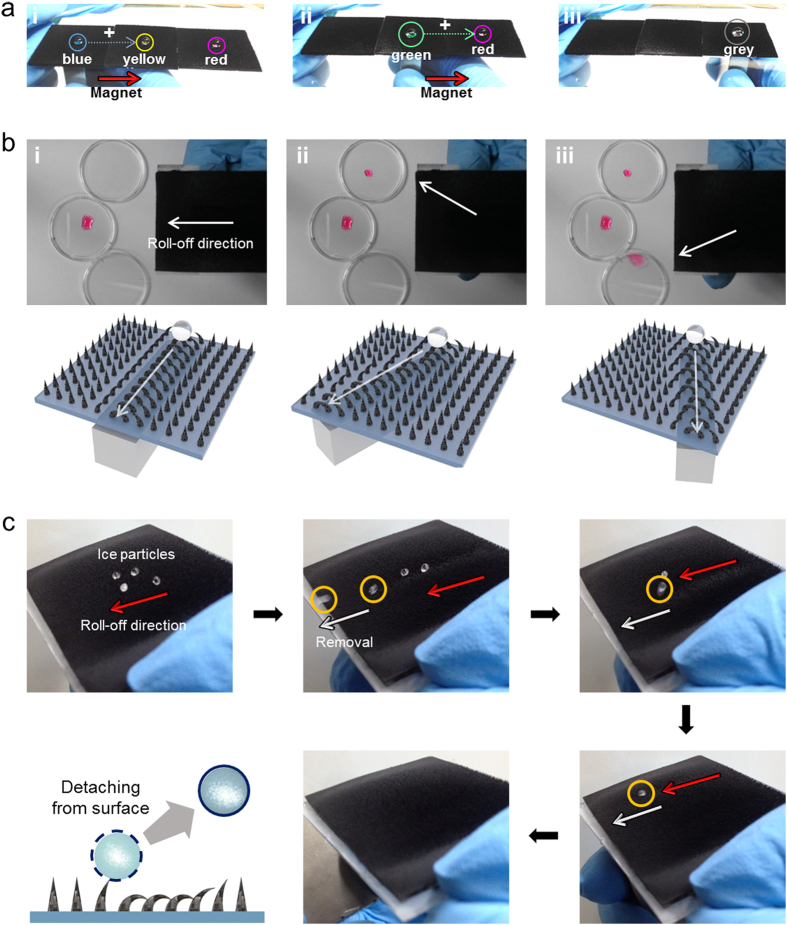
Applications of the magnetically responsive film with superhydrophobic wetting properties. (**a**) Demonstration of precise manipulation of droplets to specific targeted locations. A blue coloured droplet is manipulated with a permanent neodymium magnet to the right until it contacts the yellow droplet. After contacting the yellow droplet, the two droplets merge together, generating a larger green droplet. The merged droplet is further transported using the magnet to the red droplet, forming another merged droplet. (**b**) Guided rolling-off of the droplet to targeted locations. Pillar arrays on a magnet are flattened on the substrate along the magnetic field direction, forming an open channel on the surface. As a result, a droplet can be rolled off towards a specific target location along the channel. The channel direction can be easily tuned by rotating the magnet. (**c**) Demonstration of anti-icing properties of the film. Because of the superhydrophobic wetting properties, ice particles form with nearly perfect spherical shapes on the film. These ice particles can be removed from the surface simply by actuating the pillar arrays with a magnet.

As shown, our self-assembled pillar arrays not only exhibit magnetically responsive dynamic behaviours but also have highly stable superhydrophobic wetting properties with a nearly zero ROAs upon the application of an external magnetic field. This unique property is of considerable benefit for the active manipulation of liquid droplets. To demonstrate unique applicability, we guided the transportation of a water droplet to a specific target location using only remote magnetic force (Fig. 5a and Movie S3). Initially, 10 μl of three DI water droplets dyed with different colours (blue, yellow and red from left) were placed on the flexible film. Due to the superhydrophobicity of the film, each droplet maintained discrete spherical shapes on the film. The blue coloured droplet was moved to the right side until it contacted the yellow droplet by simply placing a permanent neodymium magnet under the blue droplet and moving the magnet to the right. The droplet maintained its superhydrophobic state during the motion with very low CAH. After contacting with the yellow droplet, the two droplets merged together, generating a larger green droplet. Similarly, the merged droplet was successfully transported to the red droplet, forming another merged droplet using just a remote magnetic field. It should be noted that this is the first demonstration of active and reversible manipulation of the position and motion of pure discrete droplets using only a remote magnetic field. So far, the active control of discrete droplets has mainly been achieved by electrowetting methods, which utilize electric fields applied to patterned electrodes. Most previous droplet manipulation techniques based on magnetic fields are limited to irreversible wetting transitions from the Cassie-Baxter state to the Wenzel state upon the bending of micro- or nanostructures without the ability to control the position and motion of discrete droplets^12^,^13^,^14^. With the dynamically actuating pillar arrays under a controlled magnetic field, our approach combines the major advantages of electrowetting (i.e., active real-time manipulation of a droplet to a specific target location) and structure-based methods (i.e., simple processes and no need for an external power source).

To demonstrate controllability over droplet motion and position, we performed an additional experiment (Fig. 5b). First, we placed the magnetically responsive flexible film on the table with a slight tilting angle of ~5°. Then, a permanent magnet was placed under the film while a water droplet dyed with red colouring was drop dispensed onto the film. Pillars under the influence of the magnetic field tilted along the magnetic field direction, forming an open channel on the magnet. As a result, the droplet rolled off towards a specific target location along the channel. The channel direction could be easily tuned by rotating the magnet (Fig. 5b and Movie S4). In addition to droplet manipulation, this magnetically responsive film with superior wetting properties is also highly useful for anti-icing surfaces. Because of the superhydrophobic wetting properties, ice particles would form with nearly perfect spherical shapes on the surface. These ice particles could be simply removed from the surface by actuating the pillar arrays with a magnet (Fig. 5c and Movie S5).

## Discussion

In the present study, we have presented, for the first time, a novel strategy that enables active, fast, precise and reversible control of the positions and motions of pure discrete droplets using only a permanent magnet on a magnetically responsive flexible film. The magnetic responsive film has randomly oriented hierarchical pillar arrays on the surface generated by the mouldless self-assembly of solutions comprising precured polymers and magnetic particles under a magnetic field. The geometries of the pillar arrays, such as diameter, height and density, can be tuned simply by controlling the magnetic field during the fabrication process. These magnetically responsive pillar arrays not only have dynamic actuating capabilities with immediate field responses and maximum tilting angles of ~90° but also exhibit stable superhydrophobic wetting properties regardless of the bending angles of the actuating pillar arrays. With these superior actuating and wetting properties, this flexible film enables active, fast, precise and reversible manipulation of discrete droplets on the surface with the use of only a permanent magnet without any additional processes or equipment. Furthermore, the fabrication of this flexible film is scalable through the simple self-assembly process without the need for any expensive processing or materials. However, our current approach has a limitation for obtaining nanoscale pillar arrays because of the size of CI particles (3–5 μm) used for the fabrication of magnetically responsive pillar arrays. Although smaller magnetic particles will allow us to fabricate smaller pillar structures, too small (<1 μm) magnetic particles will require a higher magnetic field application because of their low magnetic properties. However, smaller structures would enable the array to exhibit superhydrophobicity without the additional coating step of the array with CNPs, which will be our future work. We believe that this new magnetically responsive flexible film provides a valuable platform for active and precise manipulation of liquid droplets for a broad range of applications, from lab-on-a-chip devices for biological and chemical analyses to bioinspired functional surfaces.

## Methods

### Fabrication of magnetically responsive film with micropillar arrays

First, a base polymer of Sygard 184 (Dow Corning Korea, Seoul, Korea) and hexane (Sigma Aldrich Korea, Yongin-si, Korea) were mixed with a 1:1 weight ratio and vigorously stirred. Then, carbonyl iron particles (Sigma Aldrich Korea, Yongin-si, Korea) were added to the mixture with the same total weight as the PDMS base polymer. The mixture containing the carbonyl iron particles was sonicated for 30 min in a water bath. After the sonication, a curing agent of Sygard 184 was put in the solution at 10 wt% to the base polymer, and the solution was sonicated again. The composite solution was then spray-coated using a spray gun onto a cured PDMS substrate placed on a neodymium magnet (4 cm (W) ^χ^ 4 cm (L) ^χ^ 2 cm (T), maximum flux density: ~4 T, purchased from JL magnet, KOREA). The sprayed sample on the magnet was then placed in a convection oven for thermal curing for 2 h at 70 °C, which resulted in magnetically actuating self-assembled micropillar arrays. The micropillar arrays were spray-coated with 0.5 wt% CNPs (Sigma Aldrich) dispersed in 2 ml of acetone followed by 1 h drying at 70 °C, resulting in magnetically responsive hierarchical pillar arrays with superhydrophobicity.

### Simulation of the magnetic field around the magnetically responsive film on a magnet

The magnetic field around the magnetically responsive film on a neodymium magnet was simulated using Finite Element Method Magnetics software (FEMM 4.2, http://femm.foster-miller.net). The simulation was conducted for two different types of magnets (a: 4 cm (W) ^χ^4 cm (L) ^χ^ 2 cm (T), maximum flux density: ~4 T and b: 2 cm (W) ^χ^ 2 cm (L) ^χ^ 1 cm (T), maximum flux density: ~3 T) operating in air.

### Analysis of the structural change of the magnetically responsive pillar arrays under a magnetic field

The magnetically responsive structural changes and actuating motions of the pillar arrays were investigated with upright optical microscopy by controlling the position of a neodymium magnet under the magnetically responsive film. The magnetically responsive film with randomly oriented pillar arrays was attached to a flat glass slide to prevent bending of the film. The position of the film was controlled by a manual 3-axis stage, and the dynamic responses of the pillar arrays were captured with a CCD camera attached to the microscope.

### Analysis of wetting properties of the magnetically responsive pillar arrays

The static CAs and advancing/receding CAs were measured using a contact angle analyser (Drop Shape Analysis System DSA100, Kruss, Germany). The 10 ~30 μl deionized (DI) water droplets were gently placed on the magnetic pillar arrays for static CA measurement. The advancing/receding CAs were measured by smoothly increasing and decreasing the volume rate of the DI water droplet. The droplet images were captured by an optical microscope on the contact angle analyser. The ROA was determined by slowly tilting the substrate until a droplet started to roll off and recording the angle of the substrate at that instant.

### Manipulation of droplets on the magnetically responsive film

A 10 μl DI water droplet was placed on a magnetically responsive film fixed on a glass slide. A neodymium magnet was located right under the droplet. The magnet was then moved to a specific target location. As the magnet moved towards the location, pillar arrays immediately ahead of the magnet laid flat against the substrate. As a result, the droplet rolled over the bent pillars, following the magnet. The motion of the droplet was recorded using a digital camcorder.

### Analysis of anti-icing property of the magnetically responsive film

A magnetically responsive film with 10 μl of deionized water droplets on the surface was placed in a refrigerator at –10 °C for 2 hours to completely freeze the droplets. Then, a neodymium magnet was moved under the film to actuate the magnetically responsive pillar arrays. Detachment and removal of the iced droplets from the surface were captured using a digital camcorder.

## Additional Information

**How to cite this article**: Kim, J. H. *et al.* Remote Manipulation of Droplets on a Flexible Magnetically Responsive Film. *Sci. Rep.* **5**, 17843; doi: 10.1038/srep17843 (2015).

## Supplementary Material

Supplementary Movie S1

Supplementary Movie S2

Supplementary Movie S3

Supplementary Movie S4

Supplementary Movie S5

Supplementary Information
