# Supplementary material for: Remote Manipulation of Droplets on a Flexible Magnetically Responsive Film
Source: Sci Rep. 2015 Dec 9;5:17843. doi: 10.1038/srep17843 (PMC4673453; doi:10.1038/srep17843)
Supplement: Supplementary Information [file srep17843-s6.doc]

Supporting Information

Title: Remote Manipulation of Droplets on a Flexible Magnetically Responsive Film

Jeong Hun Kim^†^, Seong Min Kang^†^, Byung Jun Lee, Hangil Ko, Won-Gyu Bae, Kahp Yang Suh, Moon Kyu Kwak and Hoon Eui Jeong*


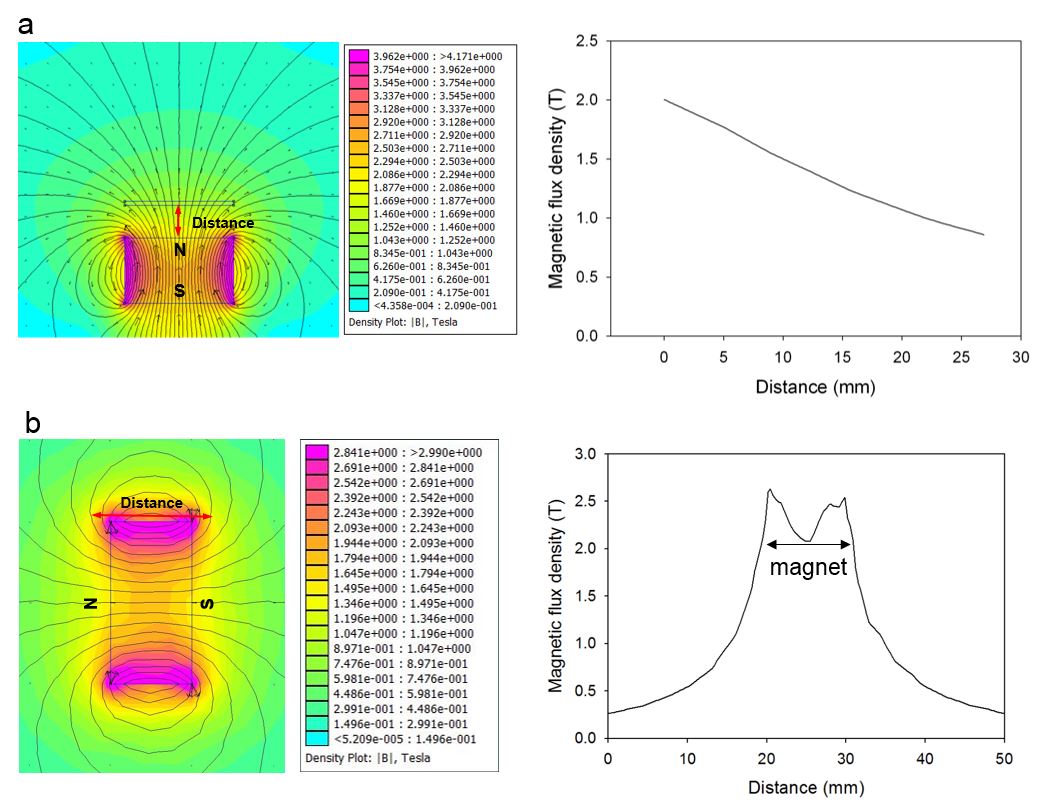


**Figure S1.** Simulation of magnetic flux density as functions of a) vertical and b) horizontal distances between the magnet and the sample.


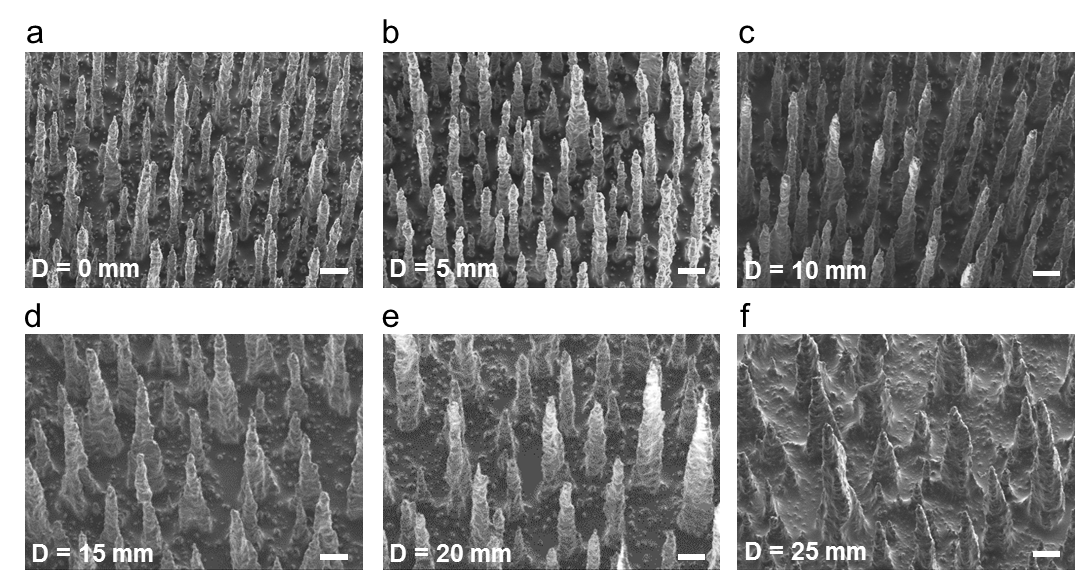


**Figure S2**. SEM images of the fabricated magnetically responsive pillars as a function of distance (D) between the magnet and the sample. a) D = 0 mm, b) D = 5 mm, c) D = 10 mm, d) D = 15 mm, e) D = 20 mm, f) D = 25 mm. Scale bars in (a-f) are 200 μm.


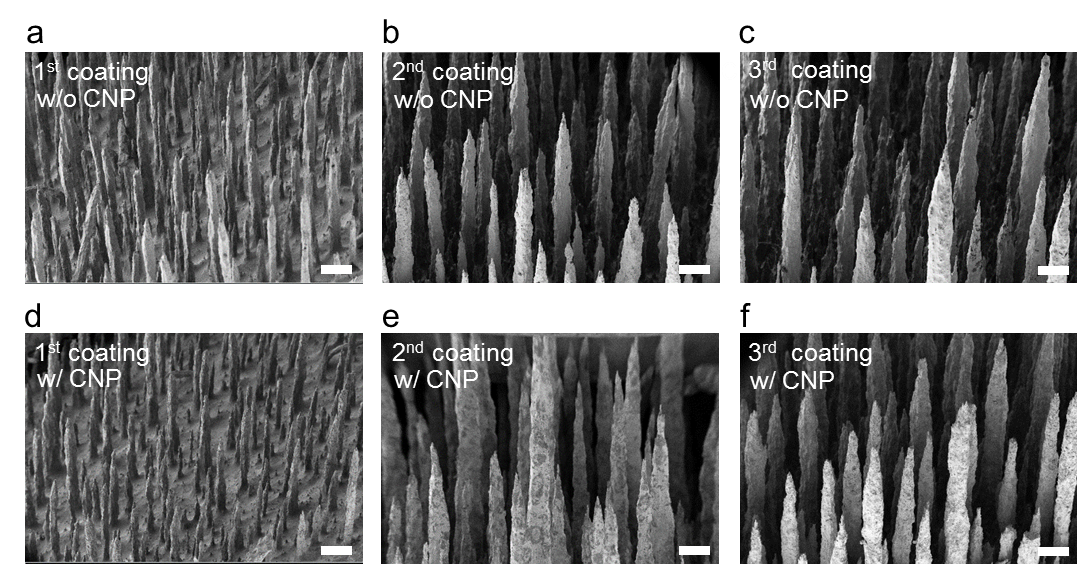


**Figure S3.** SEM images of the fabricated magnetically responsive pillars as a function of the number of coating-curing processes. (a-c) are samples without CNP coating and (d-f) are samples with CNP coating. Scale bars in (a-f) are 200 μm.


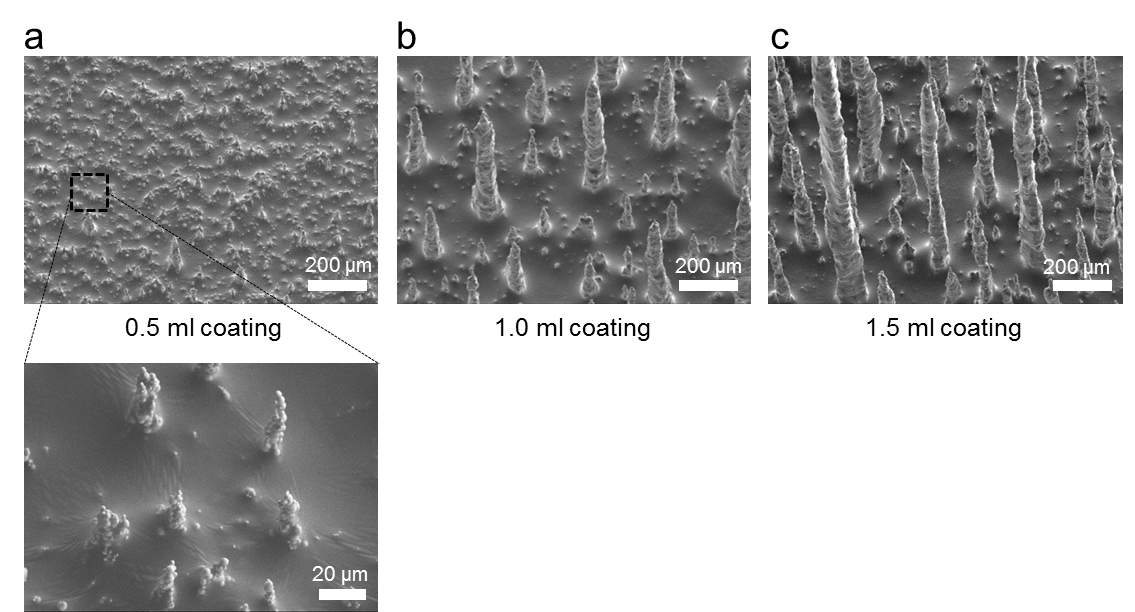


**Figure S4.** SEM images of the magnetically responsive pillar arrays prepared using different amount of sprayed mixture comprising of precured polymers and magnetic particles. 0.5 ml, 1.0 ml and 1.5 ml solutions were used for the resulting samples in a), b), and c), respectively.


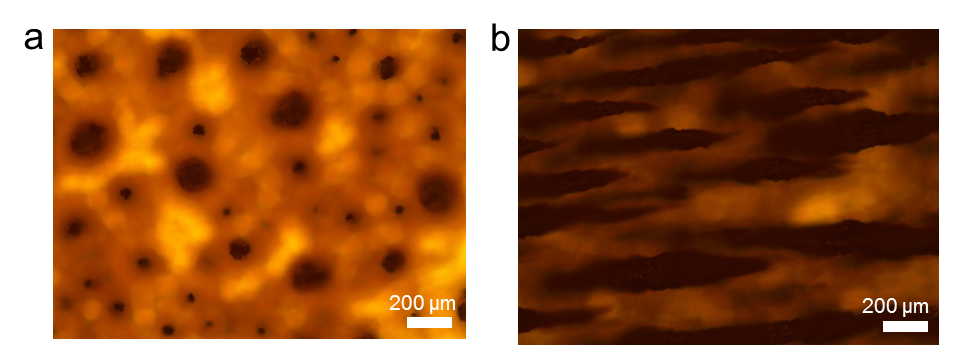


**Figure S5.** Top-views of the magnetically responsive pillar arrays a) with and b) without applied magnetic field.


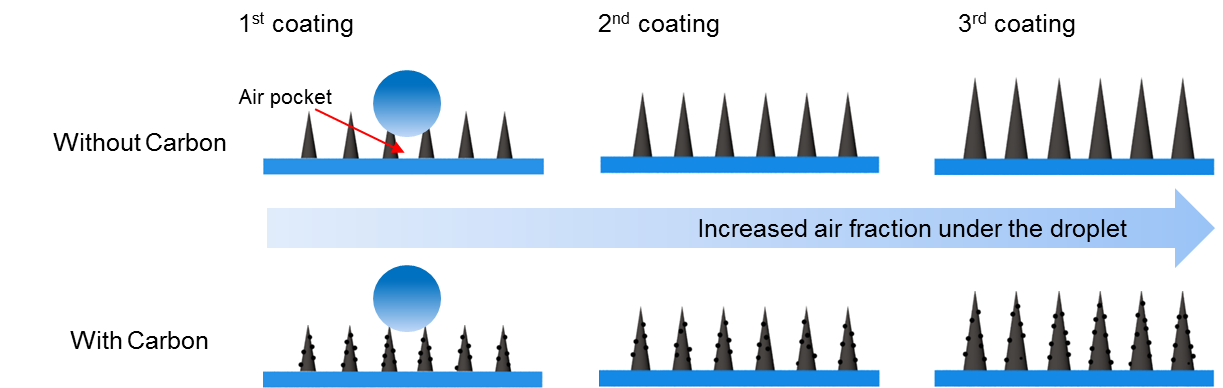


**Figure S6.** Schematic illustration of droplet wetting on various magnetically responsive pillar arrays related to the CNP coating and the number of coating-curing processes when no magnetic field is applied to the samples.


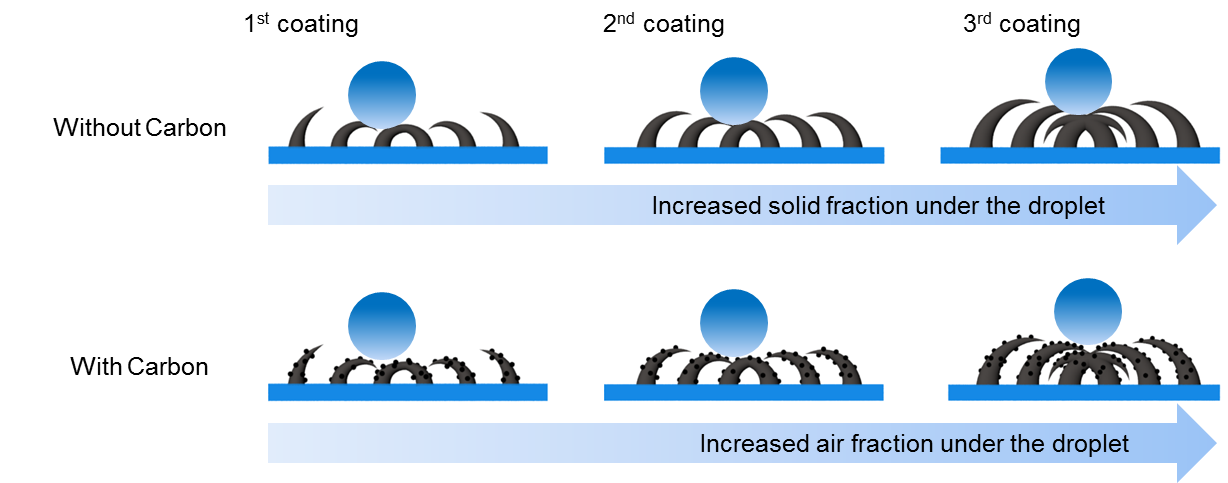


**Figure S7.** Schematic illustration of droplet wetting on various magnetically responsive pillar arrays related to the CNP coating and the number of coating-curing processes when the pillar arrays are bent by an applied magnetic field.


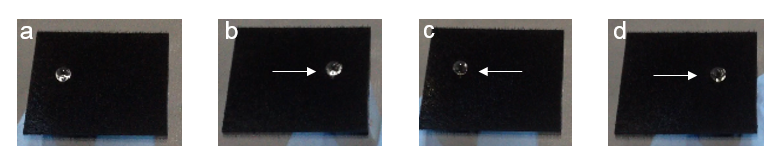


**Figure S8.** Digital camera images of reversible droplet manipulation. It is confirmed that the magnetically responsive pillars returned to the original position after removing of magnet, and the droplet manipulation can be conducted repetitively.


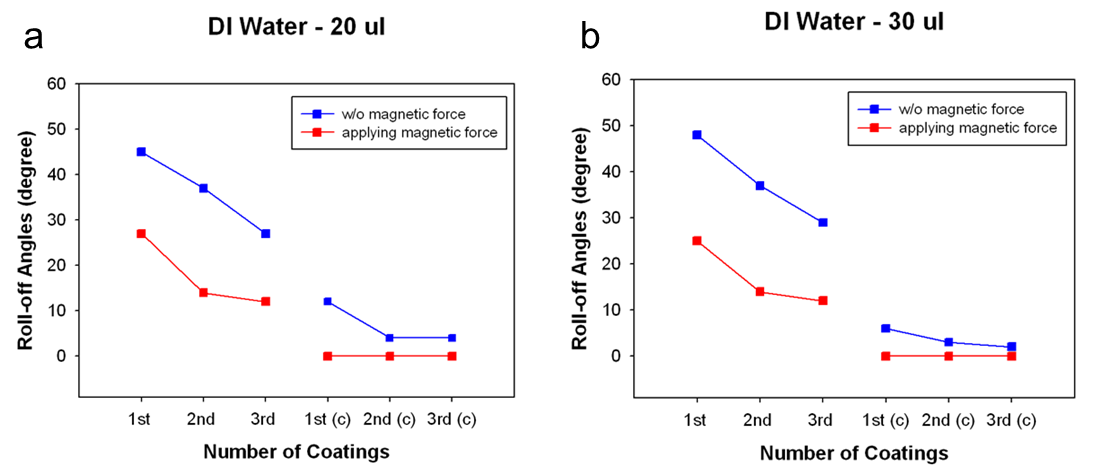


**Figure S9.** Roll-off angles of DI water with two different droplet volumes on various magnetically responsive pillar arrays for different numbers of coating-curing processes and magnetic field application. The symbol “(c)” on the x-axis of the graphs in a,b) represents CNP coated samples.

**Legends of Movies**

**Movie S1**: Active control of the dynamic structural change of the magnetic pillar arrays by an external magnetic field.

**Movie S2:** Demonstration of reversible and repetitive droplet manipulation on the magnetically responsive film with a magnet.

**Movie S3**: Demonstration of precise manipulation and mixing of droplets on the magnetically responsive film with a magnet.

**Movie S4**: Guided rolling-off of droplets to targeted locations on the magnetically responsive film with a magnet.

**Movie S5**: Demonstration of anti-icing properties of the magnetically responsive film.
